# Combination of electroporation delivered metabolic modulators with low-dose chemotherapy in osteosarcoma

**DOI:** 10.18632/oncotarget.25843

**Published:** 2018-07-31

**Authors:** Kheshwant S. Gill, Philana Fernandes, Brian Bird, Declan M. Soden, Patrick F. Forde

**Affiliations:** ^1^ Cancer Research at UCC, Western Gateway Building, University College Cork, Cork, Ireland; ^2^ Bons Secours Hospital, Cork, Ireland

**Keywords:** osteosarcoma, electroporation, metabolic modulation, cell cycle, combination treatment

## Abstract

**Background:**

Osteosarcoma accounts for roughly 60% of all malignant bone tumors in children and young adults. The five-year survival rate for localized tumors after surgery and chemotherapy is approximately 70% whilst it drastically reduces to 15–30% in metastatic cases. Metabolic modulation is known to increase sensitivity of cancers to chemotherapy. A novel treatment strategy in Osteosarcoma is needed to battle this devastating malady.

**Results:**

Electroporation-delivered metabolic modulators were more effective in halting the cell cycle of Osteosarcoma cells and this negatively affects their ability to recover and proliferate, as shown in colony formation assays. Electroporation-delivered metabolic modulators increase the sensitivity of Osteosarcoma cells to chemotherapy and this combination reduces their survivability.

**Conclusion:**

This novel treatment approach highlights the efficacy of electroporation in the delivery of metabolic modulators in Osteosarcoma cells, and increased sensitivity to chemotherapy allowing for a lower dose to be therapeutic.

**Methods:**

Metabolic modulations of two Osteosarcoma cell lines were performed with clinically available modulators delivered using electroporation, and its combination with low-dose Cisplatin. The effects of Dicholoroacetic acid, 2-Deoxy-D-glucose and Metformin on cell cycle and recovery of Osteosarcoma cells were assessed. Their sensitivity to chemotherapy was also assessed when treated in combination with electroporation-delivered metabolic modulators.

## INTRODUCTION

Osteosarcoma (OS) is of mesenchymal tissue origin and is the most common aggressive malignancy in children and young adults. It accounts for 2.4% of all pediatric malignancies worldwide. The estimated average survival rate of patients with OS following diagnosis at metastatic stage (15 to 30%) is four to five years [1, 2]. This is likely due to the high propensity of OS to haematologically metastasize early and progress rapidly given its primary sites with rich blood supply e.g. metaphysis of distal femur, proximal tibia and humerus. Current gold standard treatment involves use of high-dose neoadjuvant chemotherapies followed by *en bloc* resection of the cancer, which may include amputation in some cases. The overall survival rates in OS patients have not improved despite recent developments and advances in treatment strategies, prompting rigorous study of possible means of treating OS. Treatments for OS now include gene, targeted, and immunotherapy with progress in molecular biology [3–5].

The physiological states in cancer have resulted in complex regulatory mechanisms of cellular metabolism [6]. Cancer cells co-opt this normal regulation to fuel inappropriate cell proliferation and support survival in abnormal tissue contexts, leading to differed metabolism of tumor tissues from that of normal tissues from which cancer arises [7–9]. Cancer cells depend mainly on glucose metabolism for their energy production and macromolecular synthesis. The shift to aerobic glycolysis from mitochondrial respiration in rapidly proliferating tumor cells is a characteristic hallmark - a phenomenon known as the Warburg effect [10]. The high biomass requirements of rapid proliferating cancer cells are fulfilled by aerobic glycolysis, although it is inefficient from an energetic aspect [11]. The distinct metabolism of tumor cells makes targeting of metabolic pathways a promising approach for therapeutic interventions. Several metabolic modulators that alter essential malignant cell survival pathways have been developed with some success in recent years [12]. However, the success of metabolic modulating agents in cancer depends on a better understanding of their mechanism and identification of the ideal tumor type to target. It is also important to study these modulators as both single agents and in combination with other agents. The adequacy of treatment demographics i.e. dosing and schedule, tumor type and treatment response evaluation remain uncertain although these drugs have been tested in clinics. Glucose analogue 2-deoxy-D-glucose (2DG) used in renal cell carcinomas resulted in dose-limiting toxicities such as fatigue, sweating, and prolonged corrected QT (QTc) interval in electrocardiography (EKG) [13–15].

To a large extent, neo-adjuvant chemotherapy in OS has resulted in limb-salvage surgery replacing conventional amputation. Having said that, there is no consensus on whether neo-adjuvant chemotherapy improves the long-term prognosis of patients. Only 60% of OS patients respond to chemotherapy. The efficacy of these routinely used single chemotherapeutic agents in the treatment of OS (based on histological type) had plateaued. Resistance to chemotherapy could also be due to intrinsic chemotherapeutic resistance developing prior to chemotherapy as well as acquired resistance occurring after several cycles of treatment, which led to the introduction of double chemotherapy agents in the treatment of OS. The current treatment protocol in OS includes a cocktail of chemotherapeutic agents e.g. Cisplatin, Doxorubicin, Ifosfomide and an addition of high-dose Methotrexate. This first-line therapy is indicated in primary or metastatic disease states, and also as neoadjuvant or adjuvant therapies. Neoadjuvantly, the regular dose for Cisplatin given continuously as an infusion via intravenous route for 24 hours is 100 mg/m^2^, in addition to boluses of Doxorubicin for three days [16]. An essential aspect of OS management includes considering the toxicities from these chemotherapy agents and their side effects such as ototoxicity and/or hearing loss, myelosupression and risk of neutropenic sepsis or hemorrhage, ammenorhea, infertility, nephro- and cardiotoxicity, peripheral neuropathy and second malignant neoplasms (carcinogenesis). Reducing the chemotherapy dose concentrations and their complications in OS treatment is an important goal that will require the development of other treatment options and improved antidotes for the active anti-OS drugs. A novel strategy that efficiently inhibits growth and metastasis of OS is highly warranted.

Electroporation (EP) is a physical method of electrical application that allows permeabilization of cell membranes. This allows and facilitates the uptake of ions and compounds into cells across the cell membranes. A benefit to this approach is that a lower concentration of compounds can be used to achieve a similar if not better effect on cells or tumor. Clinically available metabolic modulators such as Dichloroacetic acid (DCA) are negatively charged molecules, whilst Metformin and 2DG are neutral. Irreversible electroporation (IRE) has been shown to be an effective technique for ablating human metastatic OS [17, 18]. There is no data to show the delivery of metabolic modulators using reversible EP in the treatment of cancers, particularly OS.

The combined treatment comprising of metabolic modulators and chemotherapy could be an intriguing approach for the treatment of patients with OS. Understanding the potential benefits of combining these two treatment modalities could be important in developing optimal treatment strategies for patients with advanced OS. Currently, no prospective data on this combination treatment is available. In this study, we examined the morphologies, cell cycles and effects on recovery and proliferation, and survival of OS cells treated with different metabolic modulators (delivered using reversible EP) in combination with low-dose Cisplatin.

## RESULTS

### Morphology of osteosarcoma cells post EP optimization

The murine K7M2 cell line and the human Saos2 cell line were maintained at their active growth phase and their morphological changes evaluated post EP at different field intensities. Voltage ranges used were 0 to 1.25 kV/cm for both cell lines, with an increment of 0.25 kV/cm between groups. After EP, cells were cytospun and stained to study its cellular morphology at 24 hours.

**Figure 1:**
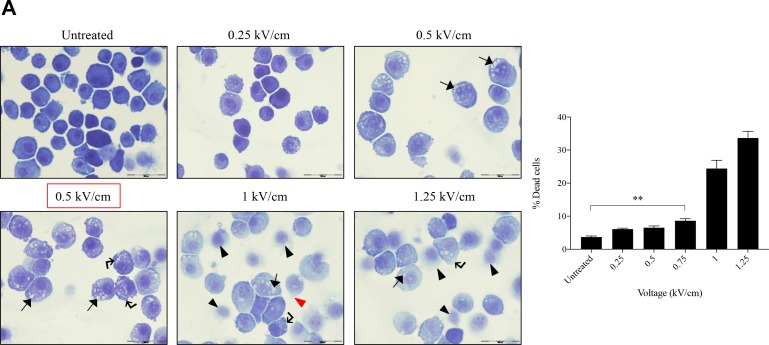

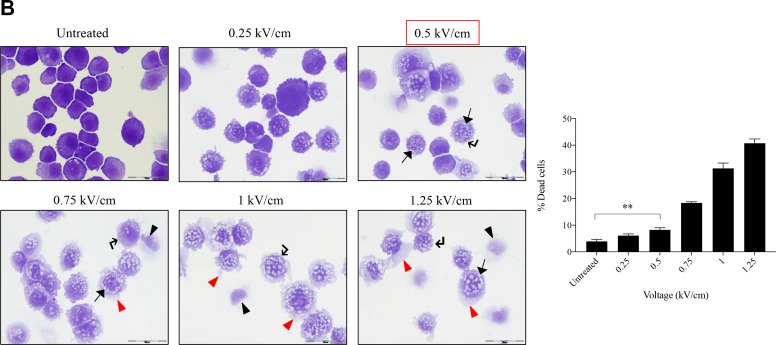

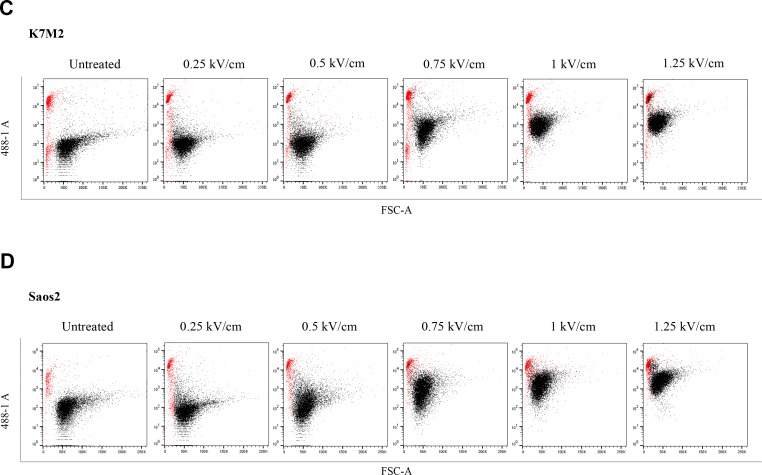

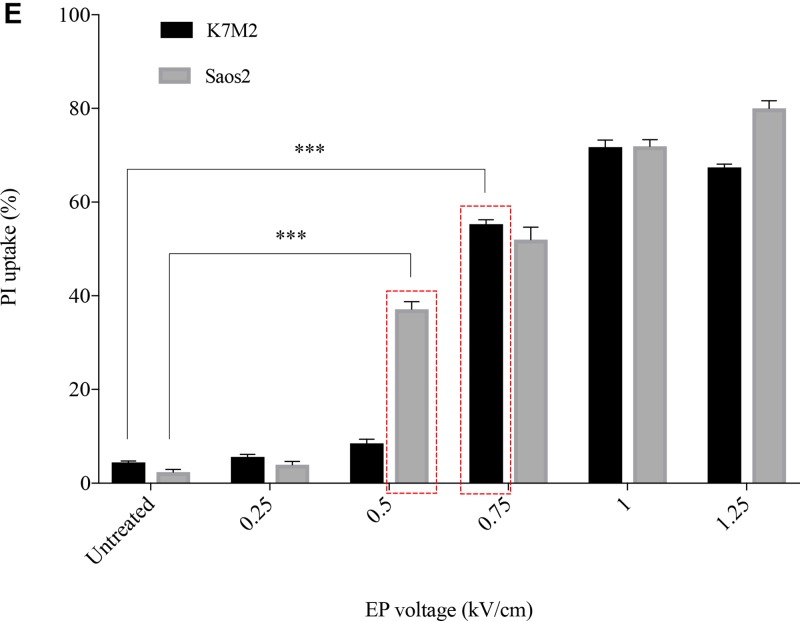

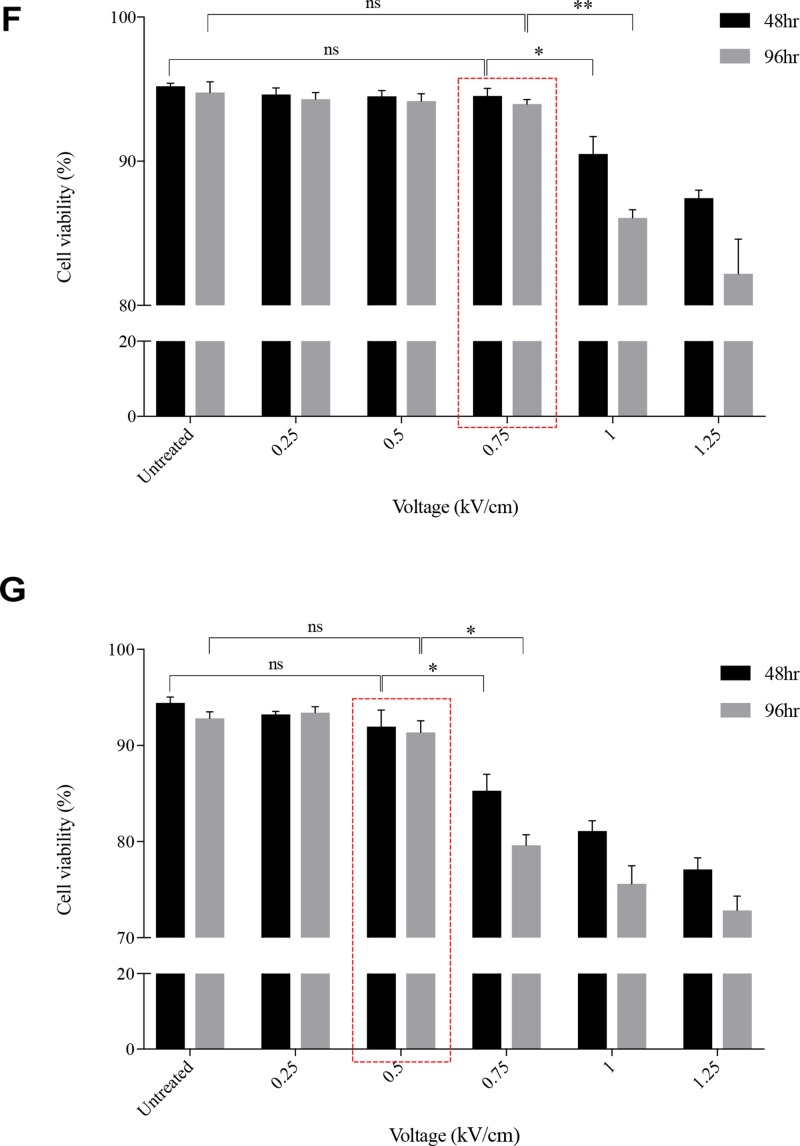
Morphology, PI uptake and viability of K7M2 and Saos2 cell lines post EP optimization Both cell lines were electroporated with eight 99 us pulses with a voltage-to-distance ratio ranging from 0.25 to 1.25 kV/cm. Cells were rested for thirty minutes prior to seeding in 6-well plates, harvested at 24 hours then cytospun and stained for morphological evaluation of (**A**) K7M2 and (**B**) Saos2 cells. Black arrows indicate intracytoplasmic vacuoles, black triangles show dead cells, right-angled arrows show blebbing of cellular membrane, and red triangles show cytoplasmic material leakage. Morphology images are representative of at least three independent experiments. Data also presented as bar graphs of percentage of dead cells post treatments. ^*^statistically significant difference in the percentage of dead cells treated with metabolic modulators for 24 hours, with and without using EP (*p* < 0.05), ^**^p < 0.01. EP efficiency was represented by population shift of viable cells (represented by black dots on dot plot) in (**C**) K7M2 and (**D**) Saos2, along Y-axis on flow cytometry dot plots, also plotted as bar graph in (**E**) for both cell lines. ^*^statistically significant difference in the percentage of PI uptake of viable cells electroporated at different voltages (*p* < 0.05), ^***^p < 0.001. Viability of (**F**) K7M2 and (**G**) Saos2 cells were measured by flow cytometry at 48 and 96 hours post EP at different voltages. ^*^statistically significant difference in the percentage of viable cells post EP at different voltages (*p* < 0.05), ns = no significance, ^*^p < 0.05, ^**^p < 0.005. Untreated = untreated baseline control cells. Red dashed boxes indicates the optimal voltage for each cell line.

Figure 1 presents the ultrastructural morphological changes of K7M2 (Figure 1A) and Saos2 (Figure 1B) OS cell lines at 24 hours post EP, at different field intensities (represented by voltage) (range of 0 to 1.25 kV/cm). No significant morphological changes were observed in the untreated groups in both cell lines. Both untreated groups showed well-rounded, smooth and regular-surfaced cells. As higher voltages were applied, more intracytoplasmic vacuoles (arrow) were observed alongside increase in size of the swollen cells. Blebbing of cellular membranes (right-angle arrow) of cells in both groups also were observed at higher voltages. Voltages of above 1.0 kV/cm for K7M2 and 0.75 kV/cm for Saos2 showed leakage of cytoplasmic material (red triangle) as a result of irreversible EP causing rupture of cellular membranes. There were more ‘ghost-like’ (black triangle) appearances of dead cells seen in field intensities higher than 0.75 kV/cm for K7M2 and 0.5 kV/cm for Saos2. Red boxes indicate the optimal voltages of reversible EP for each cell line. These voltages were derived from the combination of morphological changes, viability and PI uptake of cells post EP.

### Propidium iodide uptake and viability post EP

EP at different voltages (0 to 1.25 kV/cm) were applied to both K7M2 and Saos2 cells in the presence of a non-permeant dye, propidium iodide (PI). The efficiency of EP was evaluated by the penetration of PI, and uptake across the cell membrane using flow cytometry. Figure 1C and 1D present flow cytometry dot plots of K7M2 & Saos2 cells respectively that were electroporated in the presence of PI at ascending voltages. The red populations (dots) represent non-viable cells, whilst the black populations (dots) are viable (Figure 1C, 1D). It is evident that both OS cell lines take up more PI at higher EP voltages. However, there was a slight decrease in PI uptake of viable K7M2 cells at 1.25 kV/cm compared to 1 kV/cm, but a higher count of non-viable cells at this voltage. Both cell lines showed a direct proportional increment of PI uptake with increased EP voltages, with the highest uptake at 1 kV/cm for K7M2 and 1.25 kV/cm for Saos2 cells (Figure 1E).

There were a higher number of dead cells observed at 1 kV/cm based on morphology of K7M2 cells (black triangle, Figure 1A), and decreased viability over 96 hours (Figure 1F). Increased cell death at this voltage was due to IRE caused by high voltages from EP. Morphologically, there were fewer dead K7M2 cells at 0.75 kV/cm compared to 1.25 kV/cm (Figure 1A). Viability of K7M2 cells at 0.75 kV/cm was over 90% at 48 hours (Figure 1F). Therefore, we concluded the optimum voltage/field intensity was 0.75 kV/cm for K7M2 (red box Figure 1F) based on a combination of morphological changes, viability of cells and PI uptake. 0.75 kV/cm was the voltage used for all subsequent experiments involving K7M2 cells.

Saos2 cells also showed a direct proportional increment of PI uptake as higher EP voltage were applied, with the highest uptake at 1.25 kV/cm (Figure 1E). However, there were fewer viable Saos2 cells observed morphologically above 0.5 kV/cm (Figure 1B), therefore making it the optimum voltage (red box Figure 1G) for reversible EP, allowing PI to enter the cells and yet maintaining their viability at 90% over 96 hours (Figure 1G). 0.5 kV/cm was the voltage used for all subsequent experiments involving Saos2 cells.

### Effects of metabolic modulators delivered using EP relative to modulators alone

**Figure 2:**
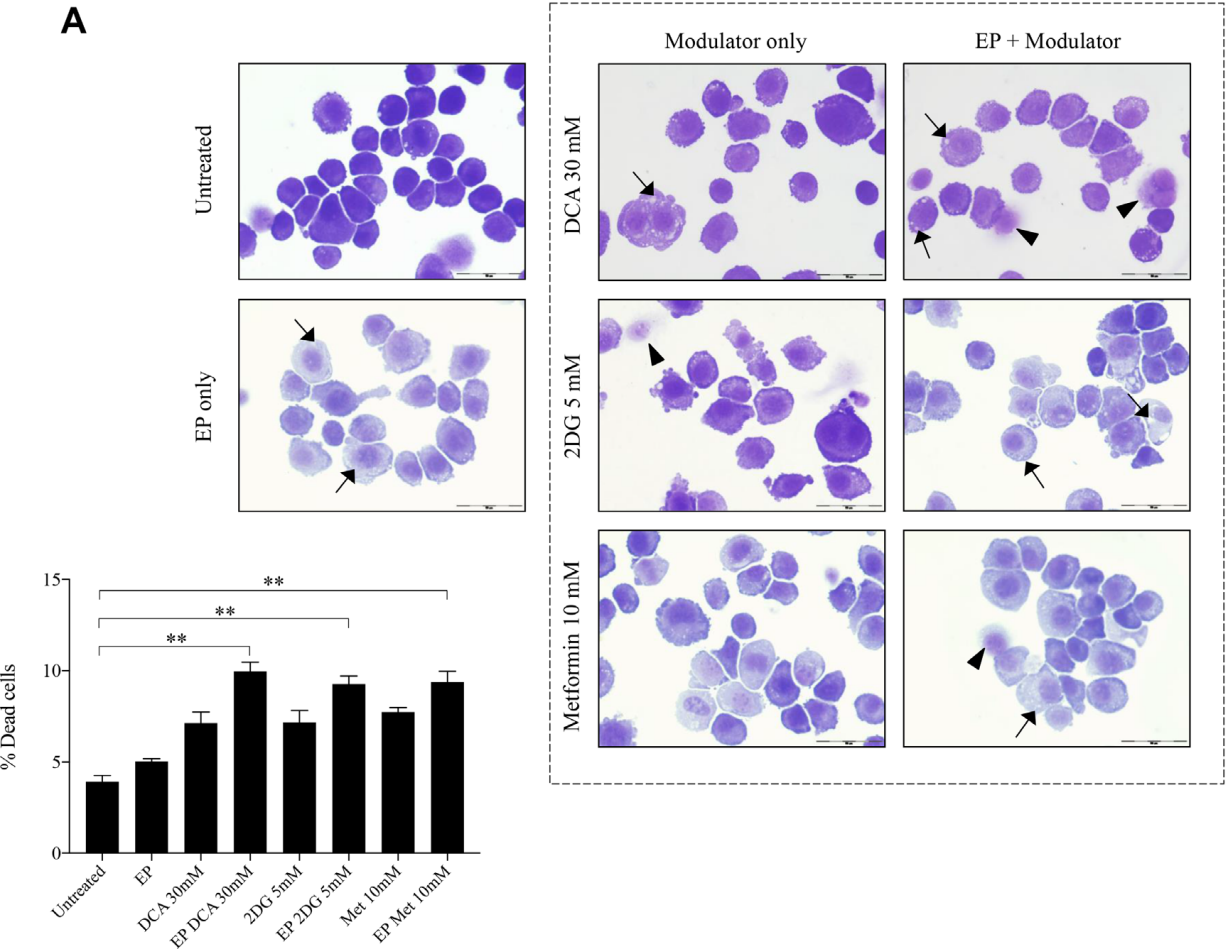

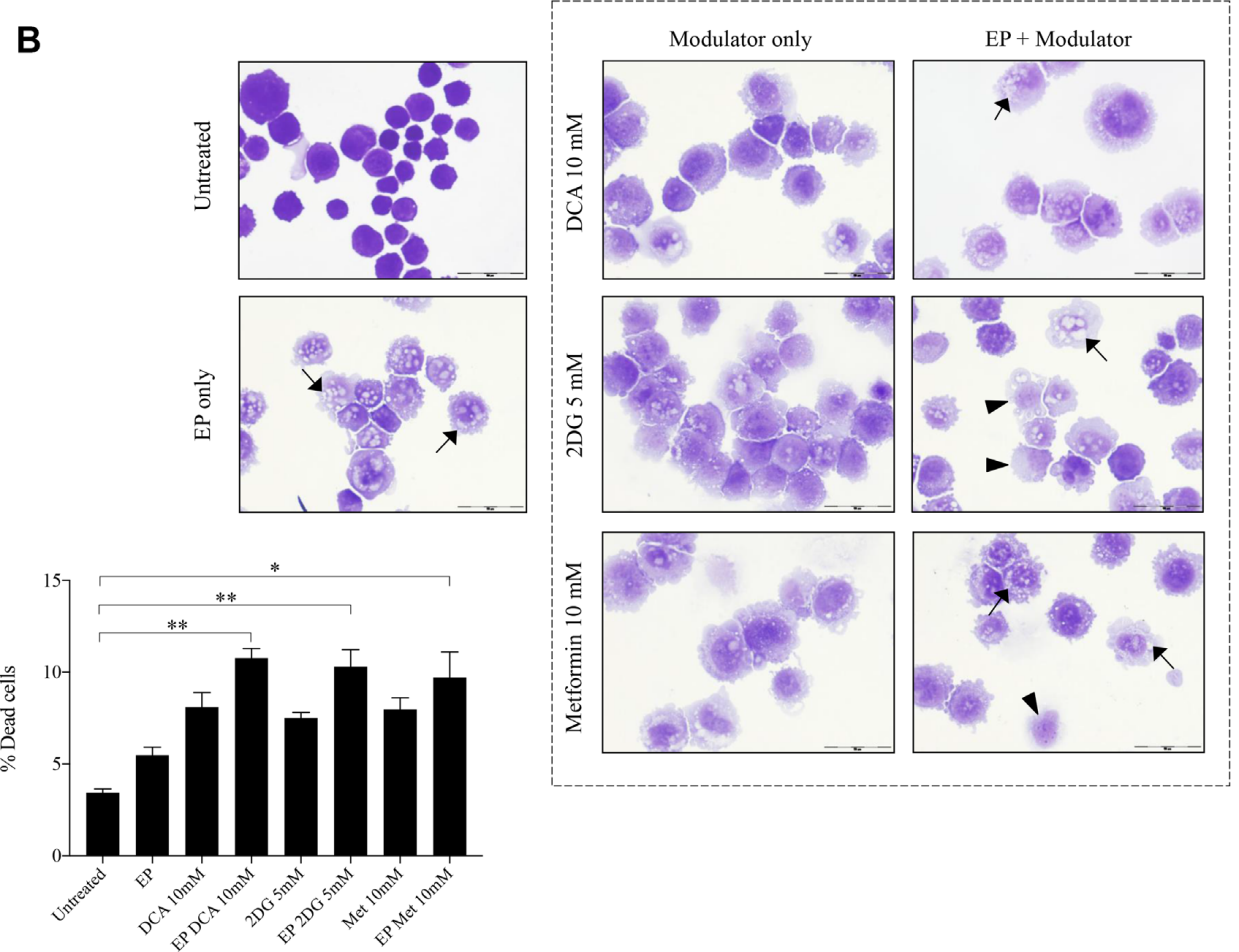

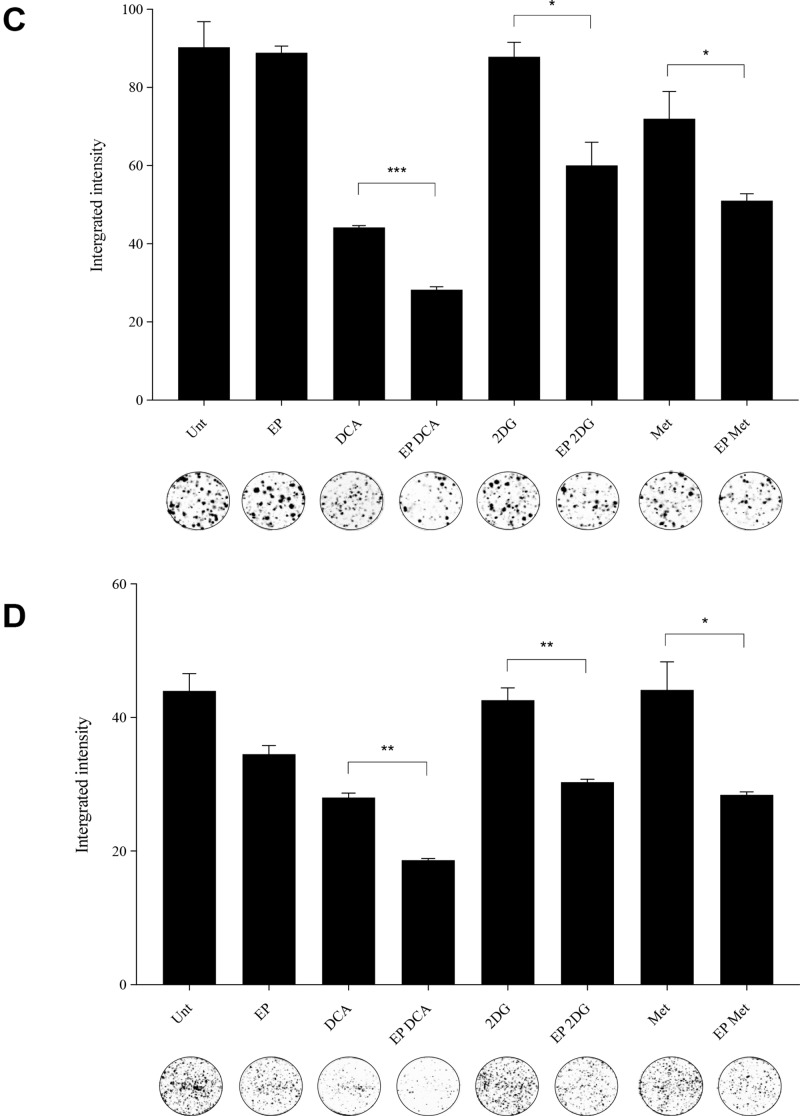
Morphology and recovery of K7M2 and Saos2 cell lines treated with metabolic modulators delivered using EP relative to modulators alone Morphologies of (**A**) K7M2 and (**B**) Saos2 cell lines at 24 hours post EP in the presence or absence of different metabolic modulators at their optimized concentration. Data also presented as bar graphs of percentage of dead cells post treatments [(A) and (B)]. ^*^statistically significant difference in the percentage of dead cells treated with metabolic modulators for 24, with and without using EP (*p* < 0.05), ^**^p < 0.05. The effects of metabolic modulators delivered with and without using EP on recovery of (**C**) K7M2 and (**D**) Saos2 cell lines were quantified by fluorescent intensities of colony formation over a period of time. Each well shown is a representative image of at least nine similar wells (three independent experiments). Data also presented in bar graphs as mean integrated intensity ± SEM of three independent experiments. ^*^statistically significant difference in the number of colonies formed when cells were allowed to recover post metabolic modulator treatments delivered with and without using EP (*p* < 0.05), ^*^p < 0.05 ^**^p < 0.005 and ^***^ *p* < 0.001. Unt = untreated baseline control.

The optimum concentrations of the candidate metabolic modulators were obtained by a series of dose responses on OS cells, treated actively (with EP) and passively (without EP). There were notably higher numbers of dead K7M2 (Figure 2A) and Saos2 (Figure 2B) cells seen morphologically in groups treated with actively delivered metabolic modulators for 24 hours (optimized concentration). Dead cells were defined morphologically as cells that had lost their plasma membrane integrity leading to the loss of cell’s identity, cell fragmentation, or engulfment by adjacent cells [19]. In these two figures, the black arrows show intracytoplasmic vacuoles as a result of EP, and black triangles show dead cells. In the EP-delivered treatment groups for both cell lines, there was a slight increase of total dead cells. The ability of K7M2 and Saos2 cells to recover and proliferate after a 24-hour treatment with metabolic modulators delivered actively was significantly reduced compared to the groups treated with modulators alone (Figure 2C, 2D), assessed by colony formation assay. Active delivery of DCA was most effective in inhibiting the recovery in both cell lines. Data was statistically significant with values of *p* < 0.05 throughout all treatment groups. These findings suggest an increased uptake of metabolic modulators when delivered actively by EP, allowing them to exert their potency on OS cells more effectively i.e. halting their cell cycle allowing for reduced rate of proliferation. We conclude that delivering metabolic modulators using EP negatively affects the ability of OS cells to recover and proliferate over a period of time.

### Effects on cell cycle by metabolic modulators in combination with EP

**Figure 3:**
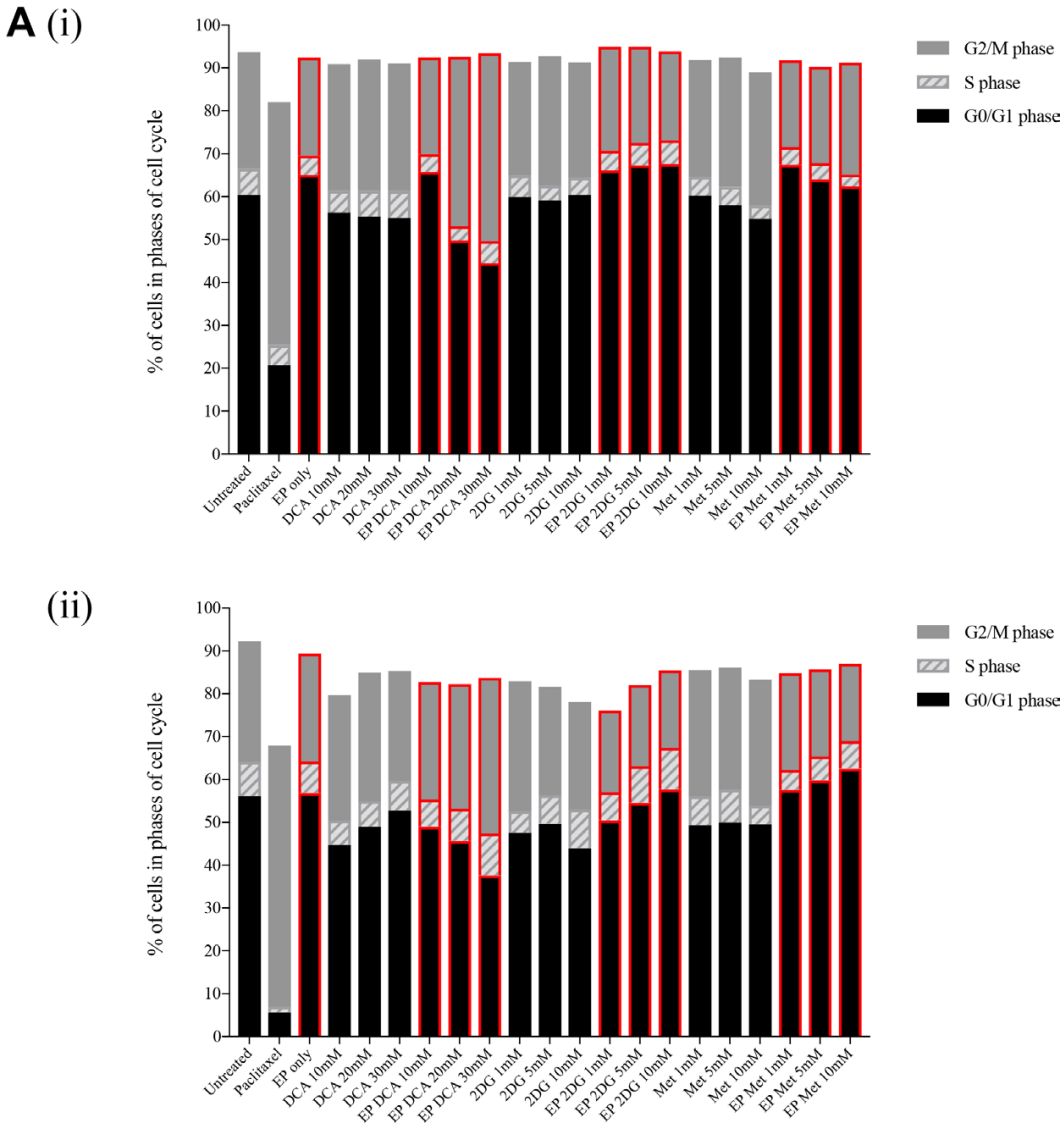

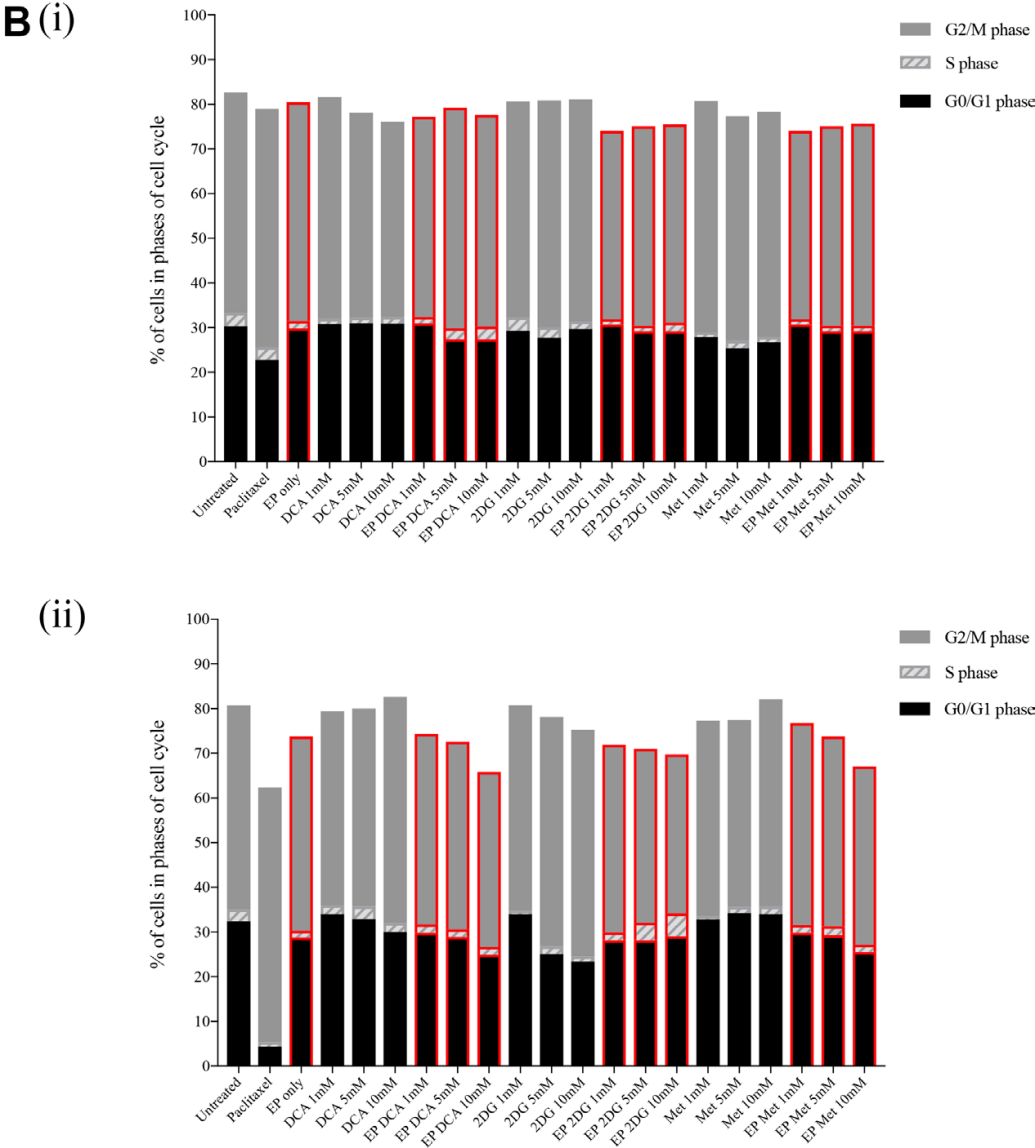

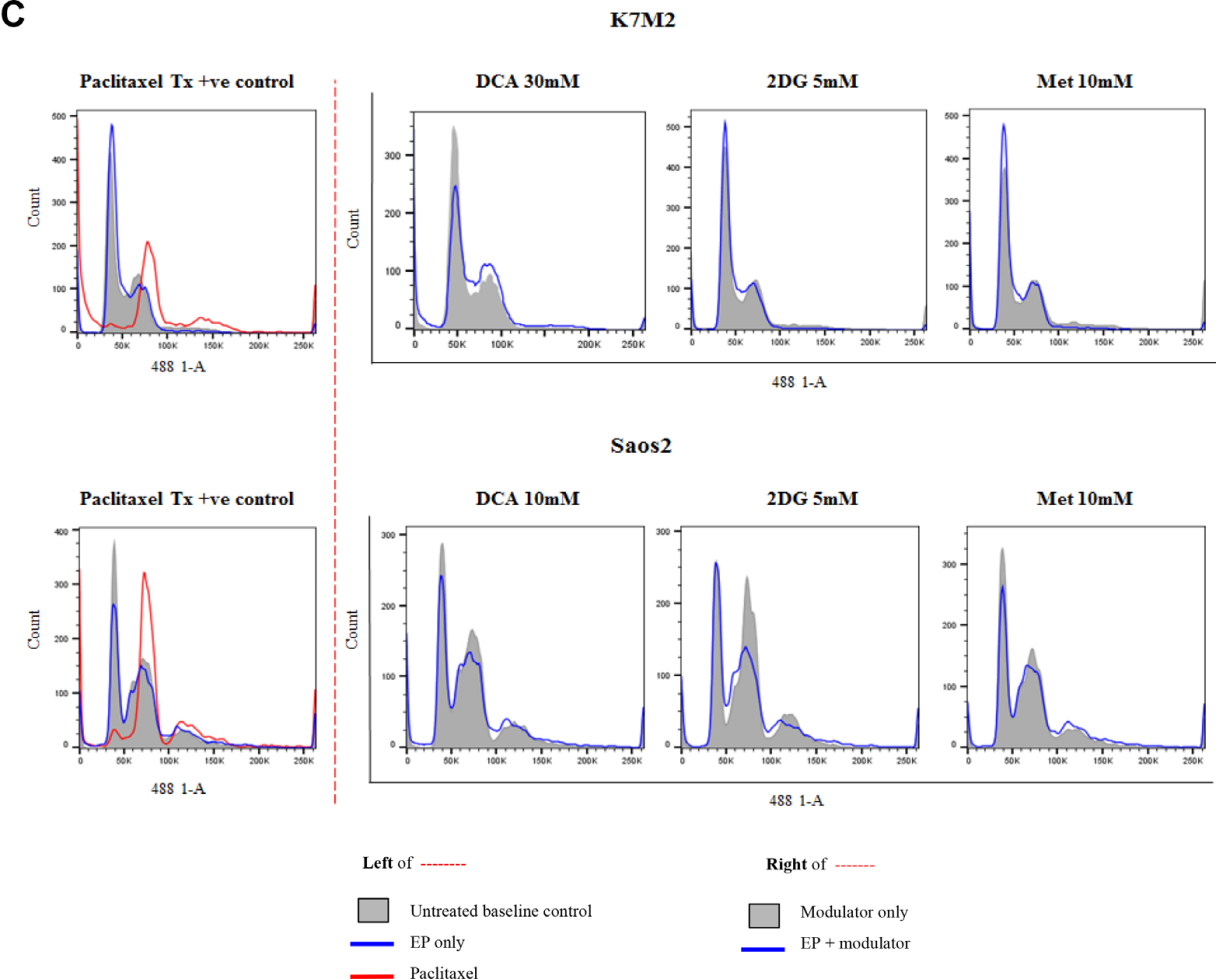
Effects of metabolic modulators on cell cycle of both OS cell lines Cell cycle phases at 8 hours K7M2 [**A**(i)] and Saos2 [**B**(i)], and 24 hours K7M2 [A(ii)] and Saos2 [B(ii)] were analyzed and quantified after fixing and staining both cell lines with PI. The DNA content were analyzed using flow cytometry. Bars with red borders in (A) and (B) represent actively (using EP) treated groups. In (**C**), the control group histograms (left of broken red line) are as follows: grey tint – untreated baseline control, red line – positive control, blue line – EP only group. Cell cycle effects post treatment with metabolic modulators at the optimized concentrations delivered actively or passively were analyzed and their representative histograms are shown in (C) for K7M2 and Saos2 cell lines. Histograms on the right of the broken red line: grey tint – passive treatment, blue line – active treatment.

In order to assess the effects of these clinically available metabolic modulators in combination with EP, we looked at their cell cycle effects on both K7M2 and Saos2 cells. Both cell lines were treated passively and actively for 8 and 24 hours at the following concentrations - DCA at 10 mM, 20 mM and 30 mM for K7M2 and 1 mM, 5 mM and 10 mM for Saos2 cells, 2DG and Metformin at 1 mM, 5 mM and 10 mM for both cell lines. Paclitaxel 5 uM was used as a positive control in this experiment. Figure 3C presents representative histograms of K7M2 and Saos2 cells treated with metabolic modulators at the optimized concentrations, passively (grey tinted) and actively (blue line) for 24 hours (right side of broken red line). Control groups (untreated baseline control, EP only, and Paclitaxel 5 uM) are shown on the left of the broken red line (Figure 3C).

Cell cycle analysis was performed at 8 and 24-hour time points. 8-hour treatment with metabolic modulators showed little inhibition [Figure 3A(i) and 3B(i)]. However, at 24 hours EP-delivered DCA showed a pronounced inhibition of K7M2 cells at G0/G1 phase and increased G2/M phase compared to the groups that were passively treated [Figure 3A(ii)]. There was also an increment in the S phase of K7M2 cells in the EP DCA group [Figure 3A(ii)], suggesting failure of progression of these cells from the S to G2/M phases in their cell cycle. EP delivered DCA also showed reduction in G0/G1 and G2/M of Saos2 cell cycle phases. The G2/M phase of this cell line were halted at higher concentrations of DCA [Figure 3B(ii)]. K7M2 cells treated with 2DG using EP also showed stalling of their cell cycle at the G0/G1 phase at higher doses, causing a lesser degree of replication of these fast-growing cells due failure of progression to G2/M phase. In the Saos2 group, it was observed that EP delivered 2DG had reduced in the G2/M phase compared to those treated passively [Figure 3B(ii) and 3c]. EP delivered Metformin in the K7M2 cells showed little reduction in the G2/M phase but an increase in G0/G1 phase compared to the passively treated group, implying that these groups of cells had also failed to progress in their cell cycle. Further, a decrease in the G0/G1 and G2/M phases of Saos2 cells treated actively with Metformin was seen [Figure 3B(ii)]. On the whole, a greater effect of these metabolic modulators delivered using EP was seen in the K7M2 compared to Saos2 cells. This was likely due to the nature of K7M2 cells having a higher proliferation rate.

The optimum concentration of metabolic modulators delivered using EP were decided based on their effects on the OS cell cycles analyzed using flow cytometry and cellular morphological changes from dose responses (data not shown). We conclude that OS cells treated actively with metabolic modulators have a higher effect on halting OS cells at different phases of their cell cycle compared to passive treatment.

### Viability and morphological changes post combined treatment

**Figure 4:**
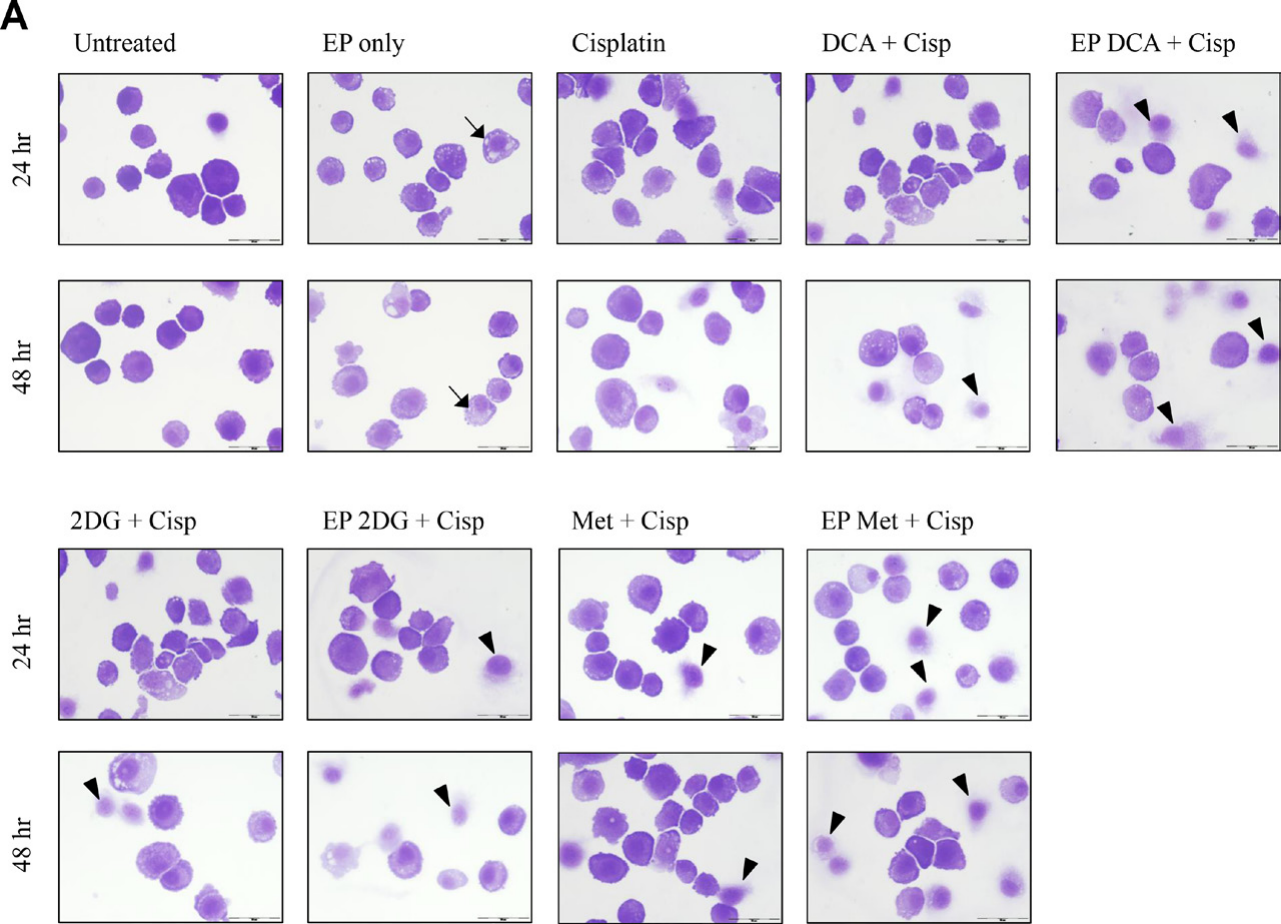

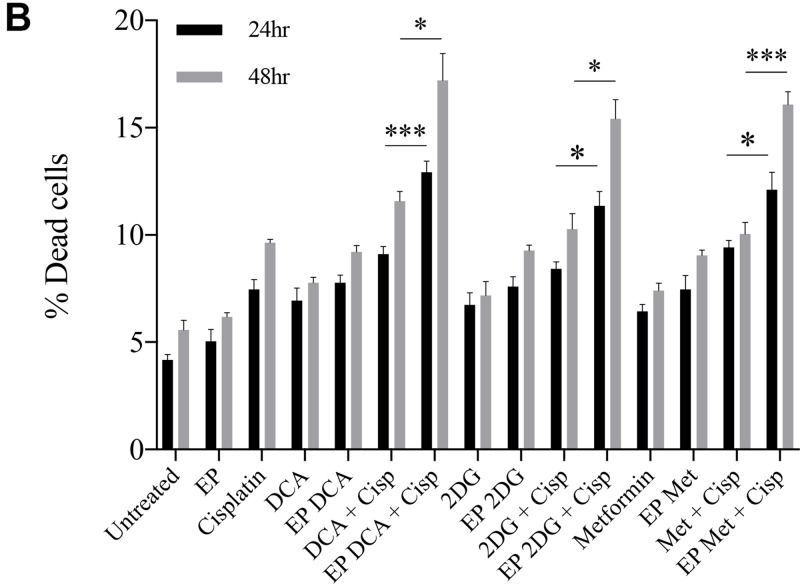

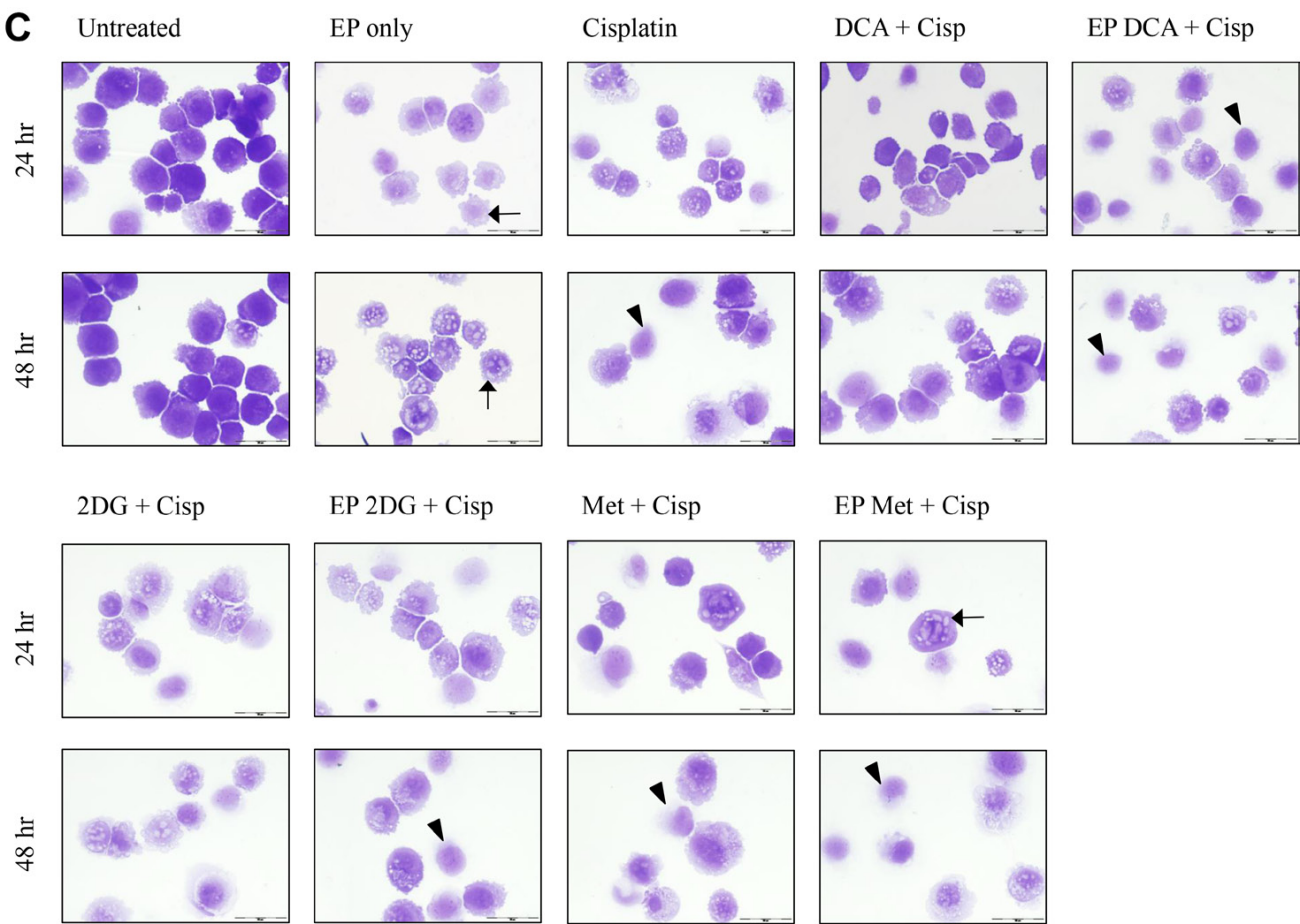

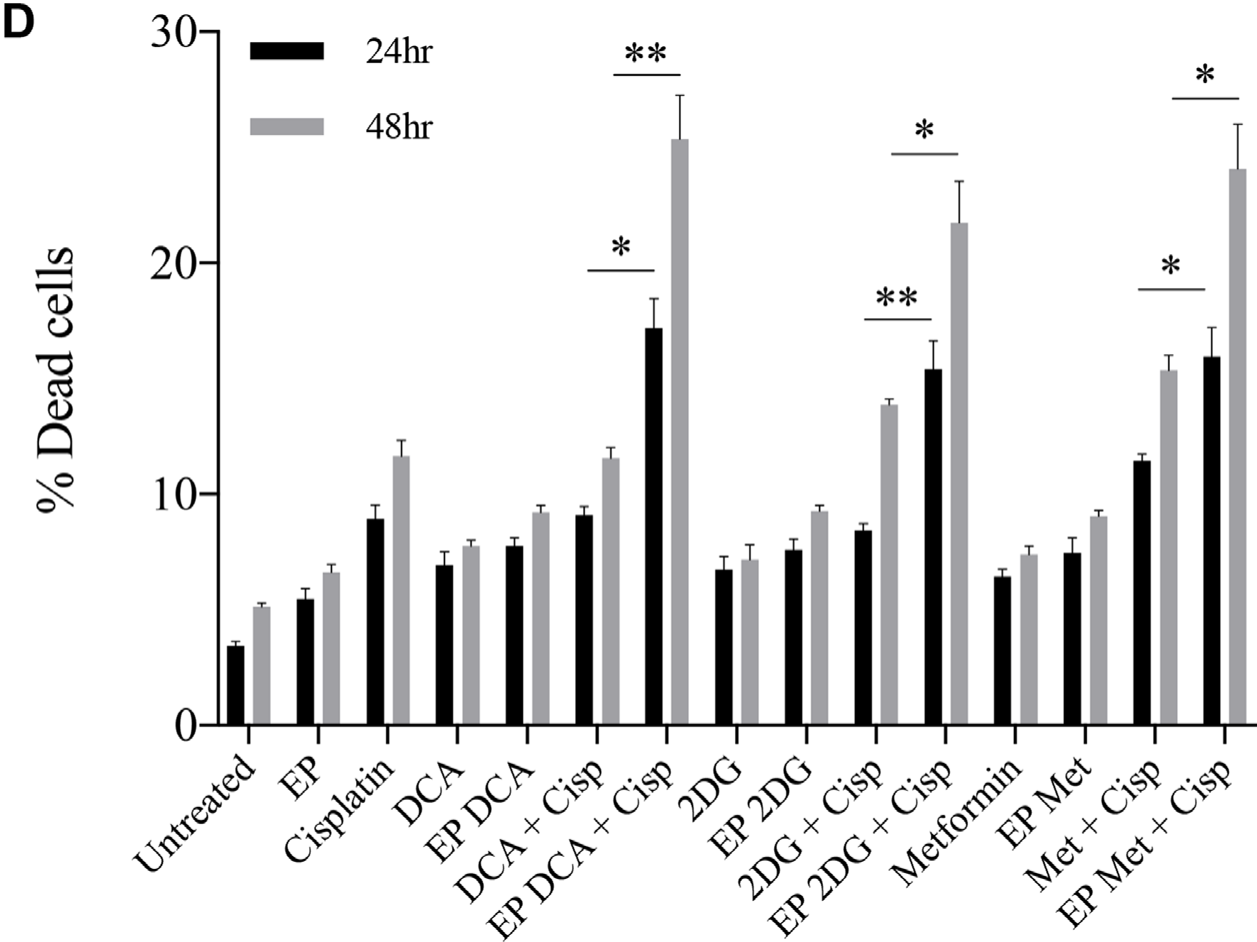

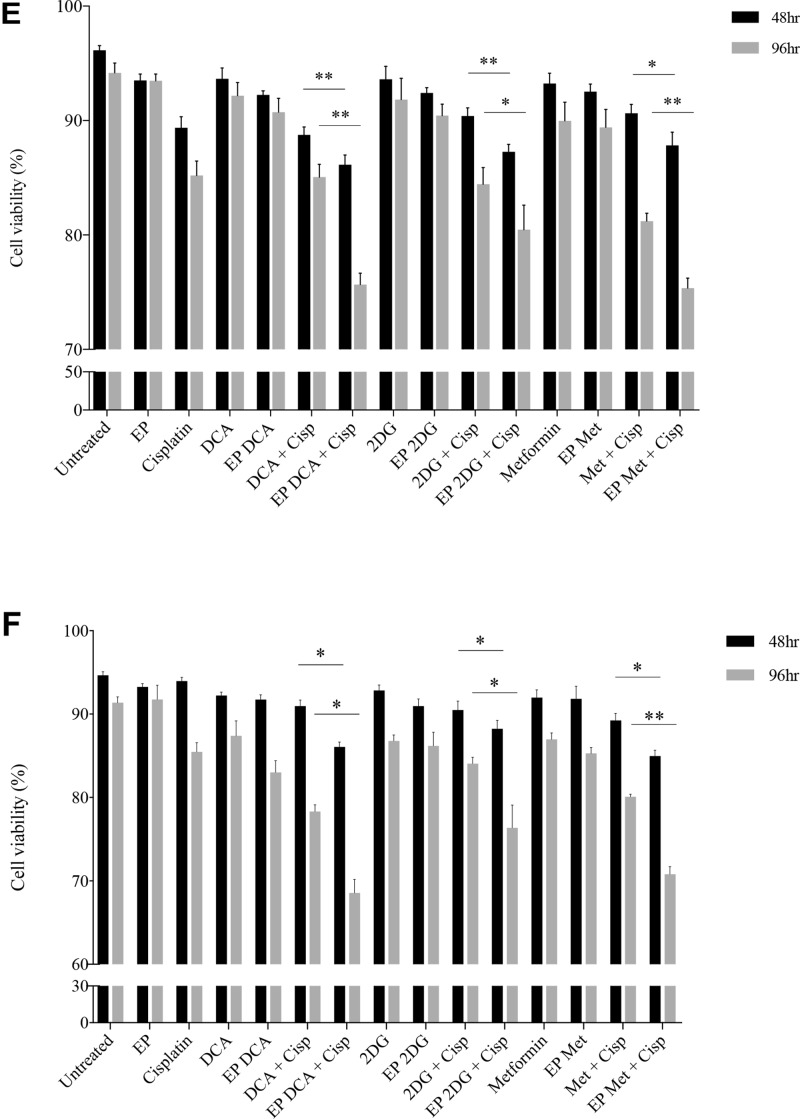

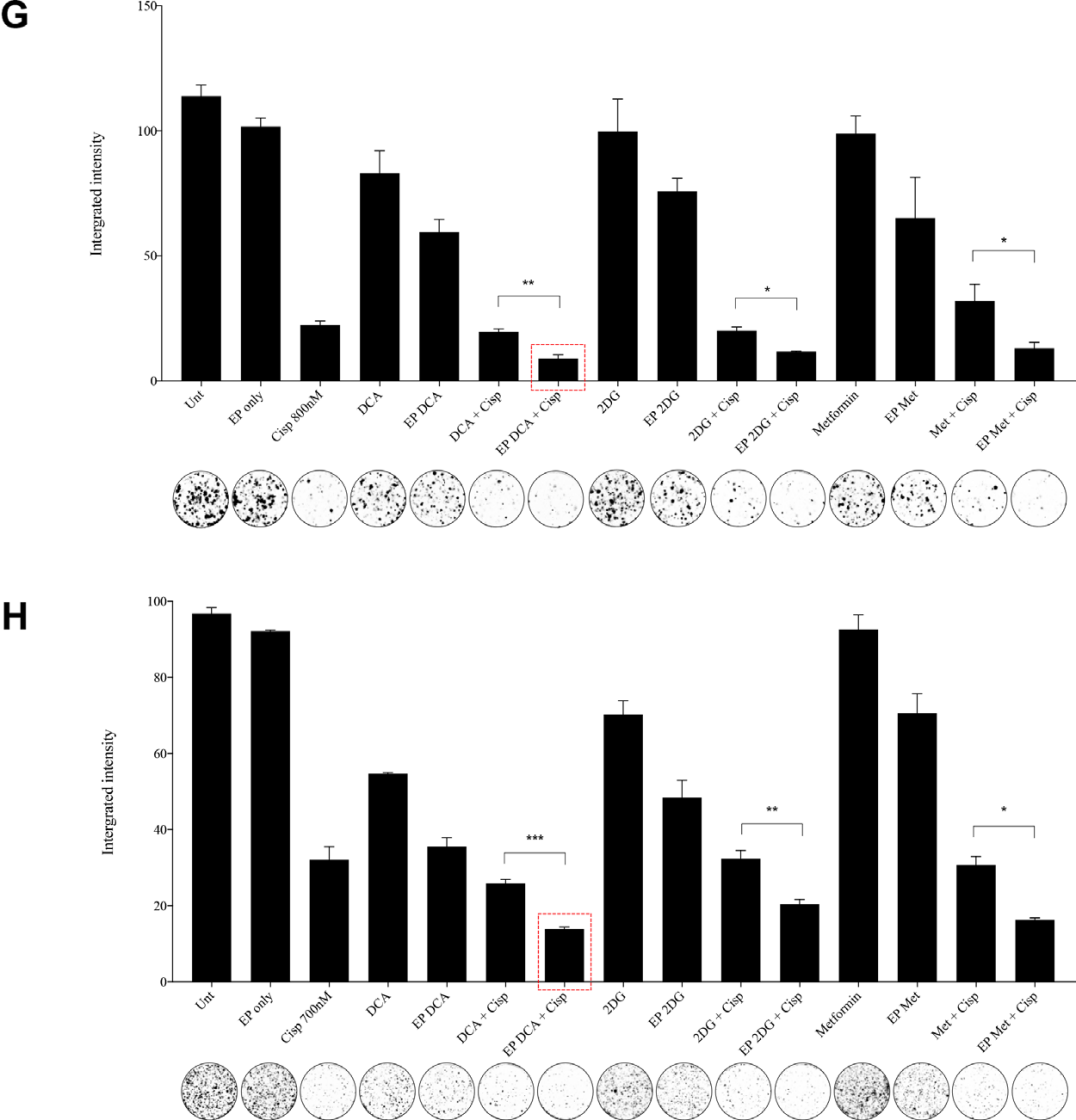
Morphology, viability and the recovery of K7M2 and Saos2 cell lines post combination treatment Morphologies of (**A**) K7M2 and (**C**) Saos2 cell lines at 24 and 48 hours post EP in the presence or absence of metabolic modulators, with and without the combination of low-dose Cisplatin. Cells were harvested at specific time points and cytospun and stained for morphological evaluation under light microscopy. Data also presented as bar graphs of percentage of dead cells post treatments [(**B**) and (**D**)]. ^*^statistically significant difference in the percentage of dead cells treated with combination treatment for 24 and 48 hours, with and without using EP (*p* < 0.05), ^*^p < 0.05, ^**^p < 0.005 and ^***^ *p* < 0.001. In (A) and (C) black arrows represent intracytoplasmic vacuoles and black triangles show dead cells. Viability of (**E**) K7M2 and (**F**) Saos2 was assessed 48 and 96 hours later by PI uptake using flow cytometry. ^*^statistically significant difference in the percentage of viable cells treated with combination treatment for 48 and 96 hours, with and without using EP (*p* < 0.05), ^*^p < 0.005, ^**^p < 0.05. The recovery of (**G**) K7M2 and (**H**) Saos2 cell lines post combination treatment was quantified by fluorescent intensities of colonies formed. Each well shown is a representative image of at least nine similar wells (three independent experiments). Data also presented in bar graphs as mean Integrated Intensity ± SEM of three independent experiments. ^*^statistically significant difference in the number of colonies formed when cells were allowed to recover post metabolic modulator treatments delivered with and without using EP combined with low-dose Cisplatin (*p* < 0.05), ^*^p < 0.05, ^**^p < 0.01 and ^***^p < 0.001.

Depletion of intracellular ATP levels in cancer cells increases their sensitivity towards chemotherapy [20]. A dose response to Cisplatin was performed on both cell lines to reach an optimum low-dose concentration. We assessed the viability and morphological changes in OS cells post combined treatment over a specific period of time (Figure 4A–4F). A reduced total number of K7M2 and Saos2 cells were seen in the combined treatment groups over 48 hours (Figure 4A, 4C). Also, there were more dead cells in these groups (black triangle). Viability of OS cells decreased over 48 and 96 hours (Figure 4E, 4F) when treated with the combined approach – delivery of metabolic modulators using EP and low-dose Cisplatin. This was more pronounced in the Saos2 cells (Figure 4F). K7M2 cells treated with EP-delivered 2DG combined with Cisplatin showed highest viability amongst the combined-treatment groups over a 96-hour period, possibly suggesting a prosurvival pathway of 2DG treated cells [21].

### Recovery and sensitivity of osteosarcoma cells post treatment with combination approach

Reversible EP has the potential to increase the uptake and potency of metabolic modulators in OS cells thus reducing their cell cycle and depleting intracellular ATP levels. Reduced ATP levels potentiate sensitivity of cancer cells to, and enhance the effects of low-dose chemotherapeutics [20, 22]. We assessed this theory in OS cells by looking at their recovery and colony formation post combined treatment. It is evident that OS cells treated with the combination approach negatively affects the ability of these cells to recover and proliferate, compared to the other treatment groups (Figure 4G, 4H). The optimum combination treatment was EP-delivered DCA combined with Cisplatin for both cell lines (dashed red box in Figure 4G, 4H). This was statistically significant (*p <* 0.05) across all combined treatment groups compared to others. The sensitivity assay was set up to determine the efficacy of low-dose Cisplatin in OS cells that were initially treated actively with metabolic modulators. The ability of OS cells to form colonies in Cisplatin-treated media over a period of time reduced in the combination treatment groups in both cell lines (Supplementary Figure 1A, 1B).

## DISCUSSION

In the present study, we used Osteosarcoma cells to evaluate the combination effect of metabolic modulators delivered using reversible EP, followed by the addition of low-dose chemotherapy 24 hours later. To our knowledge we are the first to report the delivery of metabolic modulators using reversible EP. The optimum EP delivery parameters for these cell lines were determined. Morphologically, there were fewer cells when treated with metabolic modulators actively compared to the passive groups. This was consistent with their decreased recovery and survival (colony formation) over a period of time for both cell lines. In our data, we have shown that when combined with reversible EP, DCA, 2DG and Metformin, were able to significantly halt murine and human OS cell cycles at different phases of cell cycle. There was also a decrease in viability when EP-delivered metabolic modulators were combined with low-dose Cisplatin, consistent morphologically with the increased number of dead cells in the combined treatment groups. We also observed fewer cells in the combined treatment groups suggesting an increase in sensitivity of OS cells to low-dose chemotherapy, indicating an increase in synergy between these two drug classes. Finally, the OS cells’ ability to recover and proliferate was significantly reduced when treated using the combined method thus reducing their survival.

As mentioned previously, the gold-standard therapy for OS consists of multi-agent neoadjuvant chemotherapy followed by radical surgery and adjuvant chemotherapy. Survival outcomes of patients with OS have not improved despite attempts to refine therapy efficacy e.g. via combinations of chemotherapy and radiotherapy especially in inoperable cases, and chemotherapy dose escalations [23–31]. Cell cycle alterations are excessively implicated in tumorigenesis, genomic instability and drug resistance [32–34]. Recent efforts to expand opportunities for treatment of OS through rigorous preclinical drug development, comprehensive genomic analyses, and the implementation of a histology-exclusive clinical trial model for the investigation of new agents are now beginning to generate a number of therapeutic strategies to be implemented in upcoming studies. This combination therapy of metabolic modulators and low-dose chemotherapy is not only effective in battling the survival of OS, but also less invasive as the metabolic modulators can be delivered using reversible EP intra-tumorally and followed by systemic administration of low-dose chemotherapy. We are optimistic that this new preclinical investigation on combination therapy in OS will identify a niche that can be moved forward to new trials in treatment regimes, with the goal of improving long-stagnant survival outcomes.

EP facilitates the intracellular uptake of substances that are otherwise difficult to enter cells, making it a popular technique for cell loading. This results in a higher intracellular concentration of compounds. In the treatment of cancer, EP technology has advanced tremendously leading to development of electrochemotherapy (ECT) and its increased anti-tumor effects [35]. Today, EP is used in many clinical and biotechnological applications. A clinical trial on soft tissue tumor is ongoing at the Sarcoma and Melanoma Unit of Padova. This trial extends ECT indications to patients with advanced and deep-seated cancers, where longer electrodes and bespoke insertions are used in this trial to generate an electric field tailored to the tumor mass and its margins. This is aimed at obtaining a higher local response and tumor control even on a large or deep tumor with a single treatment. Drug delivery using EP is found to be less painful [36] and may represent a favorable minimally invasive alternative to wide surgical resection, at least in a subgroup of sarcoma patients. On the basis of high efficacy of ECT [37–42], clinicians and researches are developing strategies to extend the use of EP-delivered therapy to deep seated and visceral tumors. The development of EndoVe System led to the clinical trial of endoscopic treatment of inoperable colorectal cancer in Ireland. Results from the ESOPE trial demonstrated an 85% objective response rate in solid cutaneous and subcutaneous tumor of varying histologies [43]. EP combined with Methotrexate has been employed with success in tumor-bearing mice with implanted OS cells [44]. The NanoKnife, an IRE device, was used to treat metastatic OS lung lesions [17]. IRE was also shown to be more effective in ablating the tumor than conventional treatment in OS-bearing mice [18]. To date, there is no published data on the usage of reversible EP drug delivery in OS.

One common feature of cancer cells is their ability to use glycolysis instead of oxidative phosphorylation to produce energy, despite its disease heterogeneity with diverse alterations [45, 46]. At first, a ‘faulty’ respiratory chain was thought to be the cause of these differences leading to the increase in glycolysis by tumor cells as a compensatory mechanism to this defect. But, if cancer cells (relative to normal cells) increase glucose metabolism – to form pyruvate and NADPH as a compensatory mechanism in response to Reactive Oxygen Species (ROS) formed as byproducts of oxidative energy metabolism – then inhibition of glucose metabolism would be expected to sensitize cancer cells to agents that increase the levels of hydroperoxidases (i.e., chemotherapy agents such platinum-based therapeutics e.g. Cisplatin and quinones, known to redox cycle and produce ROS [47]. The increase in synergy between metabolic modulation and chemotherapy has been validated in colon carcinoma and lymphoma in *in vivo* studies [48]. Resistance to chemotherapeutics is promoted by an increase in tumor cells’ glycolytic rate. Accumulating data also indicate that intracellular ATP is a critical determinant of chemoresistance [49]. We demonstrated this finding in our study. OS cells showed enhanced sensitivity and synergy to induce cell death, and reduced recovery and proliferation when combined with low-dose Cisplatin, suggesting the enhanced therapeutic benefit of combined therapy of metabolic modulators delivered using reversible EP with chemotherapy *in vitro*.

Hair and hearing loss, gonadal and cardiac dysfunction, and infections associated with myositis, myelosuppression and impaired renal function are the most frequent complications associated with chemotherapy. As a result, long-term follow-up is required not only to monitor the remission status but also in order to screen for and manage late effects occurring following completion of the chemotherapy regime [50]. These adverse effects of chemotherapy can be minimized with the use of lower dose chemotherapies, given the increased synergy when combined with metabolic modulators delivered using reversible EP, as shown in our data. We propose this combination therapy as a novel addition to the armamentarium of treatments in OS.

## MATERIALS AND METHODS

### Cell culturing

Two cell lines were used in the experiments, the established human OS cell line Saos2 [51] and the murine OS cell line K7M2 [52]. Both cell lines were obtained from American Type Culture Collection (ATCC), Massachusetts. Saos2 cells were maintained in McCoy 5A media (ATCC) supplemented with 20% (v/v) fetal calf serum (Sigma, F7524). K7M2 cells were maintained in Dulbeccos Modified Media (DMEM) (Sigma, D6429) with 10% (v/v) fetal calf serum. Both cell lines were supplemented with 1% penicillin/streptomycin, and were cultured at 37°C, 5% CO_2_.

### Metabolic modulators

The concentration ranges for 2DG and Metformin used in both cell lines were 1, 5 and 10 mM. DCA concentration range for K7M2 cells was 10, 20, and 30 mM whilst for Saos2 cells were 1, 5, and 10 mM.

### Electroporation protocol

Following harvesting, 1 **×** 10^6^ K7M2 and 5 **×** 10^5^ Saos2 cells were resuspended in 800 ul serum-free DMEM and serum-free McCoy 5A respectively, and electroporated in 4 mm cuvettes (VWR) in the presence of DCA (K7M2: 10, 20, 30 mM; Saos2: 1, 5, 10 mM), 2DG (1, 5, 10 mM) and Metformin (1, 5, 10 mM). The EP parameters used were as follows: 8 pulses of 99 us at a frequency of 1Hz at various voltages – 0 to 1.25 kV/cm for both cell lines using a BTX electroporator (Harvard apparatus, Model ECM 2001). After the application of electric pulses, cells in cuvettes were left to rest in the incubator for thirty minutes prior to seeding in 6-well plates for individual experiments. Parameters for each cell line were optimized for high permeabilization and low cell death from EP alone.

### Evaluation of morphology

Morphological features of cells treated with DCA (Sigma, D54702), 2DG (Sigma, D6134), Metformin (Abcam, ab146725), and Cisplatin (Teva Pharma. BV) were examined by light microscopy. Drug-treated cells (active and passive treatment) were cytospun onto glass slides and stained using Pro-Diff (Braidwood Laboratories BAPROD1-fixed and stained with buffered eosin followed by methyl thionins). Cytospin images are representative of at least three independent experiments. Dead cells were defined morphologically as cells that had lost their plasma membrane integrity leading to the loss of cell’s identity, cell fragmentation, or engulfment by adjacent cells [19].

### Propidium iodide uptake

K7M2 & Saos2 cells were applied with electrical pulses (EP) at different voltages using the above-mentioned parameters for each cell line, in the presence of PI (5 ug/100 ul). Samples were analyzed on the BD LSR II instrument for PI uptake measurement.

### Propidium iodide viability assay

PI is a small fluorescent molecule that binds to DNA but cannot passively traverse into cells that possess an intact plasma membrane. PI uptake versus exclusion can thus be used to discriminate dead cells, in which plasma membranes become permeable regardless of the mechanism of cell death, from live cells with intact membranes. Cells were seeded at 2.5 **×** 10^4^ cells/ml and 1.25 **×** 10^4^ cells/ml (K7M2) and 4 **×** 10^4^ cells/ml and 1.85 **×** 10^4^ cells/ml (Saos2), for 48 or 96-hour treatments respectively with DCA, 2DG, or Metformin, in 2 ml/well total volume. After the given time, cells were typsinized, washed in PBS and then PI (5 ug/100 ul) was added and viability was measured using the BD LSR II instrument.

### Colony formation assay

We assessed the ability of cells to recover from treatments and form colonies on a monlayer surface. Following treatments, all adherent cells were trypsinized, counted and viability determined. Of those viable cells, 4 **×** 10^2^ cells/ml (K7M2) and 5 **×** 10^2^ cells/ml (Saos2) were seeded into a 3 ml/well of a six-well plate (in triplicate). Cells were allowed to adhere and grow between 10 to 14 days. To visualize colonies, media was removed, washed with Phosphate Buffer Saline (PBS), and cells were fixed with 96% ethanol for five minutes and stained with Prodiff solution C (Braidwood Laboratories BAPROD1). Plates were scanned using the Odyssey IR imaging system (Li-Cor, Cambridge, United Kingdom) and colonies quantified. Results are presented as integrated intensity ± SEM from at least three independent experiments.

### Cell cycle analysis

K7M2 and Saos2 cells were treated with DCA (K7M2: 10, 20, 30 mM; Saos2: 1, 5, 10 mM), 2DG (1, 5, 10 mM) and Metformin (1, 5, 10 mM) for 8 and 24 hours, with and without EP delivery. Paclitaxel 5 uM was used as a positive control. At these specific time points, cells were harvested and fixed in 70% ethanol overnight at −20° C. On the following day, these cells were resuspended in 0.5 ml PBS containing 20 ug/ml PI and 100 mg/ml DNase free RNase A (Qiagen, 19101), and incubated in the dark for one hour at room temperature. Samples were then run on the BD LSR II instrument for cell cycle analysis.

### Combination assay

Following harvesting, both K7M2 and Saos2 cells were treated with DCA 30 mM (K7M2) and 10 mM (Saos2), 2DG 5 mM, and Metformin 10 mM for 30 minutes. These metabolic modulator concentrations were optimized prior to the combination assay. The actively treated group samples (using EP) were given electrical pulses (K7M2: 0.75 kV/cm; Saos2: 0.5 kV/cm) in 4 mm cuvettes (VWR) - 8 pulses of 99 us at a frequency of 1 Hz, and rested at 37°C, 5% CO_2_ for a further 30 minutes. All samples were then resuspended in respective media and seeded at 4 **×** 10^2^ cells/ml (K7M2) and 5 **×** 10^2^ cells/ml (Saos2) into a 3 ml/well of a six-well plate (repeated in triplicate). Cells were washed with PBS on the following day, and replaced with Cisplatin-treated media for reciprocating combined treated groups: 800 nM (K7M2) and 700 nM (Saos2). Cells were allowed to adhere and grow between 10 to 14 days and analyzed as per colony formation assay.

### Sensitivity assay

Sensitivity of both K7M2 and Saos2 cell lines treated with metabolic modulators actively or passively, combined with low-dose Cisplatin was evaluated by stained colonies formed in Cisplatin-treated media. 7 **×** 10^2^ cells/ml and 1 **×** 10^3^ cells/ml for K7M2 and Saos2 cells respectively were seeded into a 3 ml/well of a six-well plate (in triplicate). Cells were allowed to adhere and grow between seven to nine days. To visualize colonies, media was removed, washed with Phosphate Buffer Saline (PBS), and cells were fixed with 96% ethanol for five minutes and stained with Prodiff solution C (Braidwood Laboratories BAPROD1). Picture of each well shown is a representative image of at least nine similar wells (three independent experiments).

### Statistical analysis

Values are presented as the mean ± standard error of the mean (SEM) for three independent experiments. We performed paired *t*-test (two-tailed) statistical analysis, *p* < 0.05 was significant. Asterisks indicate the level of significance.

## SUPPLEMENTARY MATERIALS FIGURE



## Abbreviations

OS: Osteosarcoma
EP: Electroporation
DCA: Dichloroacetic acid
2DG: 2-Deoxy-D-glucose
Met: Metformin
IRE: Irreversible electroporation
PI: Propidium iodide
