# Supplementary material for: Combination of electroporation delivered metabolic modulators with low-dose chemotherapy in osteosarcoma
Source: Oncotarget. 2018 Jul 31;9(59):31473–89. doi: 10.18632/oncotarget.25843 (PMC6101145; doi:10.18632/oncotarget.25843)
Supplement: Supplementary file 1 [file oncotarget-09-31473-s001.pdf]

Combination of electroporation delivered metabolic modulators with low-dose chemotherapy in osteosarcoma

SUPPLEMENTARY MATERIALS

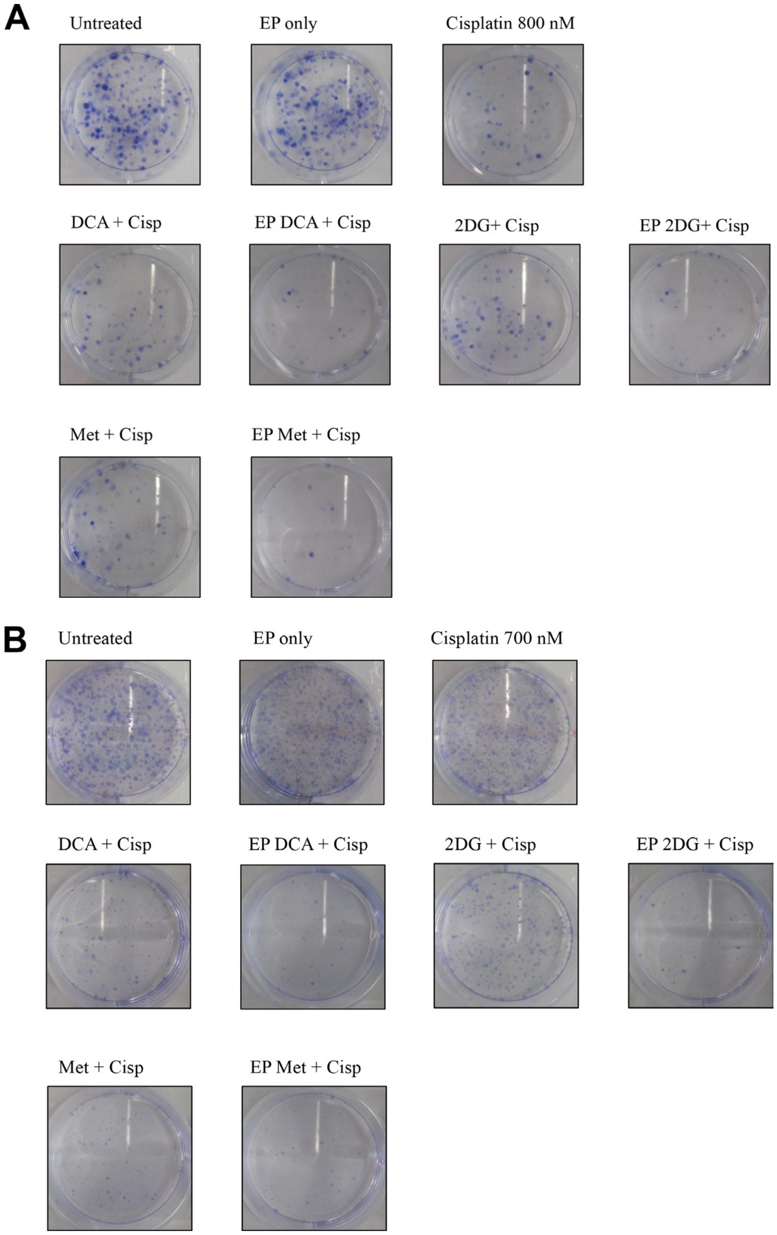

Supplementary Figure 1: Sensitivity of OS cells to low-dose chemotherapy increases when treated with metabolic modulators using EP first. Sensitivity of (**A**) K7M2 and (**B**) Saos2 cell lines treated with metabolic modulators actively (with EP) or passively (without EP), combined with low-dose Cisplatin was evaluated by stained colonies formed in Cisplatin-treated media over a period of time. Picture of each well shown is a representative image of at least nine similar wells (three independent experiments).
